# Enhancement of Biomass and Calcium Carbonate Biomineralization of *Chlorella* *vulgaris* through Plackett–Burman Screening and Box–Behnken Optimization Approach

**DOI:** 10.3390/molecules25153416

**Published:** 2020-07-28

**Authors:** Zheng Wei Chin, Kavithraashree Arumugam, Siti Efliza Ashari, Fadzlie Wong Faizal Wong, Joo Shun Tan, Arbakariya Bin Ariff, Mohd Shamzi Mohamed

**Affiliations:** 1 Department of Bioprocess Technology, Faculty of Biotechnology, Universiti Putra Malaysia, UPM, Serdang 43400, Selangor, Malaysia; jackczw@gmail.com (Z.W.C.); kavithraashree@gmail.com (K.A.); fadzlie@upm.edu.my (F.W.F.W.); arbarif@upm.edu.my (A.B.A.); 2 Department of Chemistry, Faculty of Science, Universiti Putra Malaysia, UPM, Serdang 43400, Selangor, Malaysia; ctefliza@upm.edu.my; 3 Bioprocessing and Biomanufacturing Research Centre, Universiti Putra Malaysia, UPM, Serdang 43400, Selangor, Malaysia; jooshun@usm.my; 4 Bioprocess Technology, School of Industrial Technology, Universiti Sains Malaysia, Gelugor 11800, Pulau Pinang, Malaysia

**Keywords:** *Chlorella vulgaris*, Box–Behnken Design, Plackett–Burman Design, Biomass, Calcium Carbonate, Biomineralization, Response Surface Methodology

## Abstract

The biosynthesis of calcium carbonate (CaCO_3_) minerals through a metabolic process known as microbially induced calcium carbonate precipitation (MICP) between diverse microorganisms, and organic/inorganic compounds within their immediate microenvironment, gives rise to a cementitious biomaterial that may emerge as a promissory alternative to conventional cement. Among photosynthetic microalgae, *Chlorella vulgaris* has been identified as one of the species capable of undergoing such activity in nature. In this study, response surface technique was employed to ascertain the optimum condition for the enhancement of biomass and CaCO_3_ precipitation of *C. vulgaris* when cultured in Blue-Green (BG)-11 aquaculture medium. Preliminary screening via Plackett–Burman Design showed that sodium nitrate (NaNO_3_), sodium acetate, and urea have a significant effect on both target responses (*p* < 0.05). Further refinement was conducted using Box–Behnken Design based on these three factors. The highest production of 1.517 g/L *C. vulgaris* biomass and 1.143 g/L of CaCO_3_ precipitates was achieved with a final recipe comprising of 8.74 mM of NaNO_3_, 61.40 mM of sodium acetate and 0.143 g/L of urea, respectively. Moreover, polymorphism analyses on the collected minerals through morphological examination via scanning electron microscopy and crystallographic elucidation by X-ray diffraction indicated to predominantly calcite crystalline structure.

## 1. Introduction

The advancement in today’s bioprocessing technologies has seen microalgae soared in values. Their lipids, peptides, polysaccharides, and other bioactive compounds are now the essential components in different products of cosmeceutical, pharmaceutical, and nutraceutical industries owing to their antioxidant, antihypertensive, and anti-inflammatory properties [1]. Another emerging area that could tap onto microalgal diversity originates from the biomineralogy concept of microbiological activity inducing calcium carbonate (CaCO_3_) crystals formation. The resulting crystals can either act as cementing agencies between sand grains that further increases the shear strength of soils, or by filling in the pores in-between aggregates to reduce the water conductivity [2].

Numerous microorganisms participate in the so-called microbially induced calcium carbonate precipitation (MICP) in various environments. MICP has the potential to become a sustainable source of biocement since the conventional method of producing Portland cement brings negative impacts in the form of high energy consumption and CO_2_ emissions. By comparison, MICP process occurs at ambient temperature, a stark contrast to an ordinary sandstone production which needs the temperature to be 1500 °C, and it requires a much shorter period for the full strength to develop as opposed to 28 days for standard cement [3].

Established pathways leading to CaCO_3_ precipitation include urea hydrolysis, photosynthesis, dissimilatory sulfate reduction, ammonification of amino acids, and denitrification. Biomineralization by ureolytic bacteria has received most of the attention so far, possibly due to the highest CaCO_3_ conversion efficiency as compared to other processes coupled with the straightforward and easily controlled reaction [4]. Microalgae, on the other hand, are fascinating candidates for MICP process. Besides harboring urease enzymes, they are capable of inducing biomineralization through photosynthesis pathway as well. Geomicrobiological field surveys and laboratory experiments on CaCO_3_ deposition has revealed biomineralization activity of phylum Chlorophyta, Bacillariophyta, and Cyanophyta [5,6,7].

The presence of calcium ions, dissolved inorganic carbon (DIC) (mainly in the form of ${HCO}_{3}^{-}$ ions), alkaline pH, and availability of the nucleation sites govern the MICP activity. Urea concentration may directly affect the CaCO_3_ precipitated through urea hydrolysis pathway. Nonetheless, since microalgae are the biomineralization agent in question, calcification may also proceed through ${HCO}_{3}^{-}$ and ${CO}_{3}^{2-}$ exchange during photosynthesis [8]. Most microalgae are photoautotrophs harnessing CO_2_ as their primary carbon source. Typical drawbacks of photoautotrophic cultures are usually related to the microalgae requirement for light, slow growth rate and low biomass yield [9]. 

Biomass density though significantly improved for species that can uptake other carbon sources. In this sense, mixotrophic microalgae hold more advantage against obligate photoautotrophs, benefitting from solar energy to drive the primary energy route while simultaneously able to assimilate both organic carbon and CO_2_ in the medium. This ‘photo-heterotrophy’ trait is only manifested in several species. *Chlorella*, for instance, the switch to the mixotrophic or strict heterotrophic mode when supplemented with glucose, glycerol or acetate is thoroughly established [10]. Trophic manipulation of this genus for large scale biomass and photosynthetic metabolites production have been commonly cited over the past three decades [11]. 

Besides improving biomass density, prevalent investigations on *Chlorella vulgaris* in the past seek to evaluate its feasibility toward accumulating soluble carbohydrates and lipids as raw material for biofuel applications [12]. To date, available studies on MICP process mediated by *C. vulgaris* deals with trophic comparison [13], calcification enhancement through CO_2_ sequestration in raceway pond [5], utilization of Ca^2+^ and ${HCO}_{3}^{-}$ ions from karst water [14], and co-cultivation strategy between *Chlorella*-*Sporosarcina pasteurii*, a CaCO_3_ precipitation inducer bacterium [15]. At present, no study is focusing on establishing cultural conditions that concurrently promotes substantial biomass growth and CaCO_3_ precipitation by *C. vulgaris* strain.

Design of experiment (DoE) employing statistical techniques is quite popular in many scenarios as it accorded the experimenters a facility to systematically screen for effectors, setting up predictive models and optimizing the process with a minimum number of runs. Plackett–Burman design (PBD) is a classical two-level fractional factorial method for identifying critical parameters from *N* variables running *N+1* number of experiments. On the other hand, Box–Behnken design (BBD) is a rotatable response surface technique distinguished by three-levels per factor applied to independent variables that were selected from screening exercise. This study utilized both PBD and BBD method to derive statistically fitted models describing the effects and interactions between factors that constitute the common Blue-Green (BG)-11 aquaculture medium towards enhancing biomass density and CaCO_3_ biomineralization performance of local *C. vulgaris* in shake flask system.

## 2. Results and Discussion

### 2.1. Screening of Significant Factors Through Plackett Burman Design (PBD)

Screening is a prelude to optimization experiments and is a crucial step to eliminate any insignificant factors or noise, ensuring that favorable conditions are met for the growth of *C. vulgaris* and accumulation of the product of interest. PBD is usually employed for ruggedness testing based on the multiplication of four (rather than a power of two) number of factorials which would result in small-sized runs emphasizing on determining the most significant main effects without recourse to the interaction between factors. Since the PBD option in Design Expert DoE software package allows for a minimum of eleven experimental variables, this study utilized eight-actual and three-dummy factors to initiate the screening process. PBD comprising of 14 runs were performed, which also included two center point replicates to obtain an estimate of pure error. PBD matrices showing the levels of each factor in their actual values with the corresponding biomass density and weight of CaCO_3_ precipitates are shown in Table 1.

Results from PBD experiments were then fitted to the linear first-order model as explained in the Materials and Method section (Equation (4)) with model adequacy for each of the response being subjected to analysis of variance (ANOVA). Initially, by accounting all eight factors (*A–H*) in a full model, some of the regression coefficients were found to produce individual *p*-values of >0.05. In turn, these contributed to the full model itself to be insignificant for interpreting the data. Backward elimination procedure was then carried out to exclude any unnecessary model terms one at a time, starting with the term having the highest *p*-value. Elimination stopped when only factors with *p*-values below 0.05 were left in the model, resulting in an overall significant but reduced model. 

The analysis of variance performed on the reduced models for *C. vulgaris* biomass density (Table 2) and CaCO_3_ precipitation (Table 3) has demonstrated that the models are statistically valid with *p*-values of 0.0007 and 0.0005, respectively. In other words, by looking at the corresponding *F*-value, which practically measures the ratio of between-groups variance to the within-groups variance of treatments, at 16.08 and 19.82, they would imply that both models are highly significant. There is only 0.07% and 0.05% chance that “Model *F*-value” could have occurred due to noise.

The goodness of fit of these models is indicated by the coefficient of determination (*R*^2^). The respectable *R*^2^ value for microalgal biomass density model at 0.8894, and *R*^2^ describing CaCO_3_ precipitation model at 0.9340 indicate that 88.94% and 93.40% of the variability in the two responses could be explained by the models, signifying good agreement between experimental and predicted values. Following backwards elimination results of Table 2 and Table 3, factors that posed significant effect (*p* < 0.05) towards promoting *C. vulgaris* biomass density are NaNO_3_, urea, MgSO_4_.7H_2_O and sodium acetate (*A, B, D, F*). Factors that are decidedly influencing the biomineralization of CaCO_3_ precipitates are likewise, NaNO_3_, urea, MgSO_4_.7H_2_O, and sodium acetate with an addition of exogenous factor of temperature (*A, B, D, F, G*).

**Figure 1:**
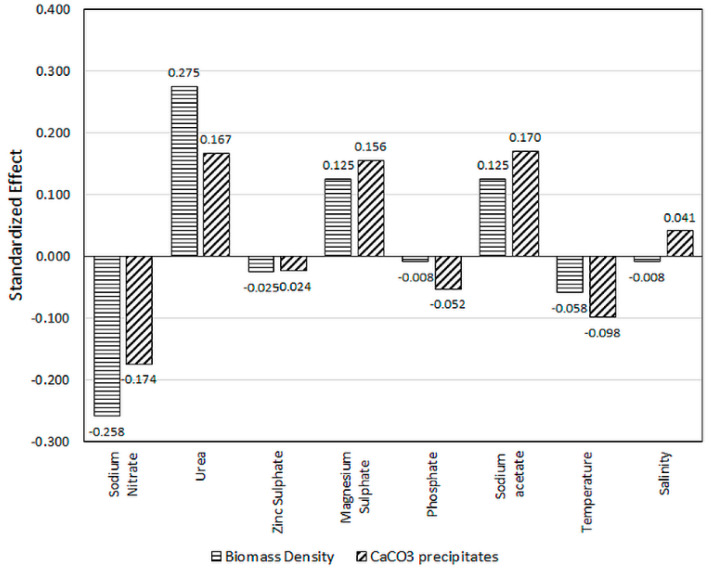
Effect of different factors on biomass density and CaCO_3_ precipitates concentration.

The positive and negative effects of each factor influencing *C*. *vulgaris* biomass and CaCO_3_ precipitation is also depicted in Figure 1. A negative value in the bar chart indicates that the shift of the factor from low level to the high level produced a decreased in biomass or CaCO_3_ precipitation, while a positive value means that the changes in a level increase such responses. Regarding the examined levels, it is apparent that urea was the most significant factor in positively enhanced microalga growth. Observation of the actual results in Table 1 points to urea providing a roughly 2.6-fold increase in biomass density when furnished with concentration ranges from 0.01 g/L to 0.1 g/L (standardized effect of 0.275), followed equally by sodium acetate and MgSO_4_.7H_2_O (both at 0.125). The highest effector that negates growth is shown to be NaNO_3_ (−0.258). Temperature (−0.058), ZnSO_4_.7H_2_O (−0.025) and K_2_HPO_4_ (−0.008) are less likely to impart significant effect on *C. vulgaris* biomass density.

The improvement in the MICP activity of *C. vulgaris* mostly paralleled the gain in biomass since all the factors in Figure 1 depict identical synergistic or antagonistic effects towards CaCO_3_ precipitation except for salinity (*H*). Based on the standardized effect values, the positive effect is mostly attributed to sodium acetate (0.170), followed closely by urea (0.167), MgSO_4_.7H_2_O (0.156) and to a lesser degree, salinity (0.041). Factors that registered in reduced biomineralization are NaNO_3_ (−0.174), temperature (−0.098), K_2_HPO_4_ (−0.052), and ZnSO_4_.7H_2_O (−0.024).

As a reduced form of nitrogen source, *C. vulgaris* affinity towards urea as compared to NaNO_3_ could be due to less energy required by the compound to undergo assimilation process, and it is potentially easier to pass through the plasma membrane of microalgae cells. Hence, lending itself towards a synergistic effect on cell growth [16]. As a viable source of carbon, acetate can be metabolized through two pathways in microalgae, the glyoxylate cycle and the tricarboxylic acid (TCA) cycle. In *C. vulgaris*, isocitrate lyase, the key enzyme for the assimilation of acetate/acetyl-CoA in the glyoxylate pathway, is synthesized constitutively. However, the glyoxylate cycle is functional only when cells are grown on acetate-containing medium [10]. During the dark period of cultivation, the activity of isocitrate lyase increases sufficiently for glyoxylate cycle to account for the syntheses necessary to maintain growth while enzymes related to TCA cycle are not induced, though the latter pathway remains active. In the event of illumination, citrate synthase, isocitrate dehydrogenase, and succinyl-CoA synthetase, which provide intermediate metabolites of the TCA cycle, are then upregulated in mixotrophic cultivation. Chlorella cells metabolizing acetate would go on to increase the incorporation of carbon into the cellular components, particularly into lipid, polysaccharide, and protein [17]. Magnesium is one of the essential macronutrients required for growth as it forms the central atom of a chlorophyll molecule for microalgal photosynthesis. It means that, for every one molecule of chlorophyll produced, one Mg^2+^ is required [18].

On the flip side, NaNO_3_ exerts a significant antagonistic effect on the biomass of *C. vulgaris*. PBD simulation in this study predicted that the biomass density gradually reduced from 1.12 g/L to 0.86 g/L with the increasing NaNO_3_ concentration in medium from 2.5 mM to 22 mM. In Soil Extract (S.E.) medium supplemented with 10 g/L glucose, Xie et al. [19] observed that nitrate had outperformed ammonium, peptone, and yeast extract, and slightly edging urea in supporting *C. vulgaris* growth. Interestingly, the authors reported that even though low nitrate concentrations (<1.00 g/L) would result in *C. vulgaris* reaching stationary phase much earlier, the growth behaviour bore a similarity to culture fed with high nitrate concentrations (≈2.00 g/L). In their study, nitrate concentration in the region of 12 mM (≈1 g/L) was suggested as critical concentration for sufficient nitrogen supply in which excess nitrogen could not be utilized by *C. vulgaris* above this limit, indicating that luxury uptake of nitrogen was not apparent.

The synergistic effect of introducing acetate and urea as supplementary carbon and nitrogen source also points to the significance of growth under alternative metabolic conditions potentially enhancing the carbonate precipitation. By looking at the concomitant increase in both biomass and CaCO_3_ precipitates, one can deduce that higher biomass would translate to higher availability of the cell surface structure for nucleation to take place. Besides the apparent increase in cell numbers, sodium acetate assimilation also produces relatively larger cells. Mixotrophic acetate feeding by *Chlorella pyrenoidosa* had evolved the cells exhibiting a mean diameter of 11.8 µm against 8.0 µm for normal photoautotrophic cells [17].

Notwithstanding that the role of mixotrophic mode upon CaCO_3_ precipitation by *C. vulgaris* was not directly tackled in the past, the effect of trophic switching in term of carbonate precipitation rate was evaluated in one study by Kawano et al. [13]. In their experiment, having utilized 5 mM CaCl_2_ and 20 mM NaHCO_3_ containing_-_medium, it was recognized that the CaCO_3_ precipitation rate of the biotic system (addition of 10^6^ cells/mL *C. vulgaris* microalga) under illumination had posted considerably higher values of 2.7–2.9 enhancement factor over baseline abiotic system owing to the photosynthetic activity of *C. vulgaris.* In contrast, the biotic system under dark condition (*C. vulgaris* grew in heterotrophic mode) registered lower biomineralization rate than those of the abiotic system, with an inhibition factor of about 0.8–0.9. The marked reduction in precipitation between the two trophic systems was related to the interaction of different organic molecules released from the cells of *C. vulgaris* with the CaCO_3_ mineral surfaces.

### 2.2. Selection of Significant Factors for Further Optimization

**Figure 2:**
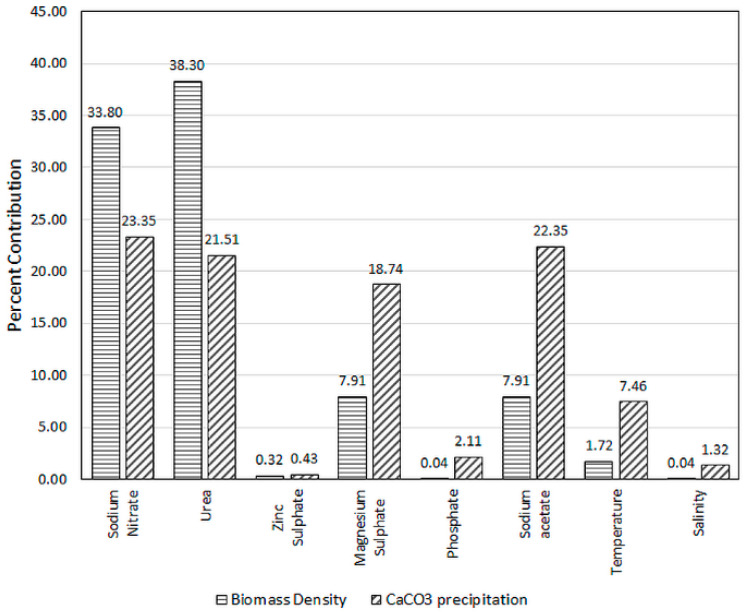
Percent contribution of different factors on biomass density and CaCO_3_ precipitates concentration.

The experimental factors were assigned in such a way that they would be simultaneously become significant to both responses. Screening criteria considered the degree of their contribution (expressed in percentages) and the corresponding statistical significances, evidencing in *p*-values of less than 0.05 from PBD results. Evidently from Table 2 and Table 3, both the biomass density and CaCO_3_ precipitates were highly affected by NaNO_3_, urea, MgSO_4_.7H_2_O and sodium acetate concentrations. The percentage contribution of the different tested factors depicted in Figure 2 shows that all four accounted for nearly 88% of the total effect exerted on biomass production and about 86% towards carbonate precipitation. Only the top three most significant factors would be chosen for further optimization; an economic decision made based on the experimental duration and availability of experimental resources. A four-factor response surface BBD design would have created 29 runs, resulting in a considerably large total runs (58) if duplication were considered for each run.

NaNO_3_ and urea were straightforward choices due to them collectively possessing 72% effect on cell growth and 45% towards MICP activity. The two medium components also exhibit explicitly small *p*-values in both ANOVA tables. Between MgSO_4_.7H_2_O and sodium acetate, both recorded similar *p*-value of 0.0428 and equally contributed to about 7.91% of individual effect towards biomass density. Nonetheless, biomineralization by *C. vulgaris* proved far sensitive to changes in sodium acetate concentration, judging from the 22.35% of effect exertion by the compound as opposed to 18.74% contribution by MgSO_4_.7H_2_O. As such, this effective rounded up the variable selection process and three factors (NaNO_3_, urea, and sodium acetate) were chosen for the second statistical step of Box–Behnken Design to realize the main effects and possible interactions among them. As the number of factors was reduced from eight to three, the concentrations of other non-significant factors were held at their respective PBD center point values in the following medium optimization study.

### 2.3. Optimization via Box–Behnken Design (BBD)

The motivation of choosing BBD instead of the more popular central composite rotatable design (CCRD) lies in the economy of experiments as it can generate fewer runs, as well as avoiding treatment combinations that may sit on the extreme points of the experiment boundaries [20]. Seventeen experimental runs with five center points were generated for the three-level BBD investigation on the combined effect of NaNO_3_ (*A*), sodium acetate (*B*) and urea (*C*) on biomass density and carbonate precipitation. Table 4 shows the experimental conditions complete with the predicted and corresponding actual results according to the factorial design. The total number of actual experiments were doubled as each standard order runs were an average of duplicates. The levels of each independent variable were determined based on the earlier PBD experiments. The center points (0) of BBD were set as equivalent to optimum values predicted by Design Expert based on PBD dataset. The previous simulation had predicted that a modified BG-11 medium composed of 9.00 mM NaNO_3_, 50.00 mM sodium acetate and 0.125 g/L urea would hypothetically maximize the target responses with desirability as high as 0.868. The low and high BBD level (designated as −1 and +1) for each of the factor was adjusted appropriately as multiples of the respective center points.

By applying multiple linear regression, data fitting of BBD results to Equation (6) from the Materials and Methods Section has generated the following correlations in terms of un-coded values:

*Cell biomass (g/L) = −2.8515 + 0.4919A + 0.0464B + 15.1944C − 0.0017AB −0.0222A^2^ − 0.0002B^2^ − 39.7778C^2^* (1)

*CaCO_3_ concentration (g/L) = −1.4309 + 0.3215A + 0.0171B + 6.4100C − 0.0193A^2^ − 0.0002B^2^ − 32.7200C^2^* (2)

The *p*-values from ANOVA analysis conducted on the second-order polynomial model terms were used as a tool to gauge the significance of each coefficient, which also indicated the strength of interaction of each parameter. Smaller *p*-values point to higher significances of the corresponding coefficients. Based on Table 5 and Table 6, both models were highly significant to describe the experiments, as indicated by *p*-values of less than 0.0008. The lack-of-fit was used as a support test for the adequacy of the fitted models. Both regression models show an insignificant lack-of-fit (*p* = 0.0770 for biomass and *p* = 0.0725 for CaCO_3_) demonstrating their insignificance relative to the pure error.

ANOVA of the biomass model shows that all the linear coefficients (*A, B, C*) proved significant. Contrary to the outcome of PBD analysis, the effect of sodium acetate is now considerably higher than the other two (*p* < 0.0015). The model term *AB* was also significant, indicating that an interaction between NaNO_3_ and sodium acetate somehow affected the growth of *C. vulgaris*. All three quadratic coefficients (*A^2^, B^2^, C^2^*) were also considered as significant. On the other hand, ANOVA of the regression model for CaCO_3_ concentration placed a remarkably high significance on urea and the least with NaNO_3_. The terms *A^2^, B^2^ and C^2^*, were equally significant to the precipitation of CaCO_3_. However, looking at both Equation (1) and (2), the quadratic terms were inferred to exert antagonistic effects on both responses. The absence of cross-product coefficients *AB, AC*, and *BC* (*p* > 0.05) may lead to the assumption that factors have an additive effect on the CaCO_3_ precipitation.

### 2.4. Mutual Effects of Factors on Biomass Density and CaCO_3_ Concentration

**Figure 3:**
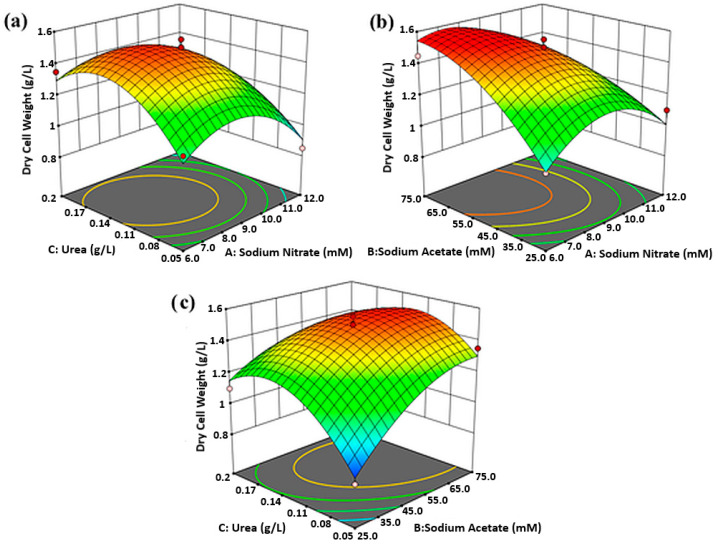
Response surfaces and contour plots showing the mutual effect of (**a**) urea and NaNO_3_, (**b**) sodium acetate and NaNO_3_, (**c**) urea and sodium acetate on *C. vulgaris* biomass density.

**Figure 4:**
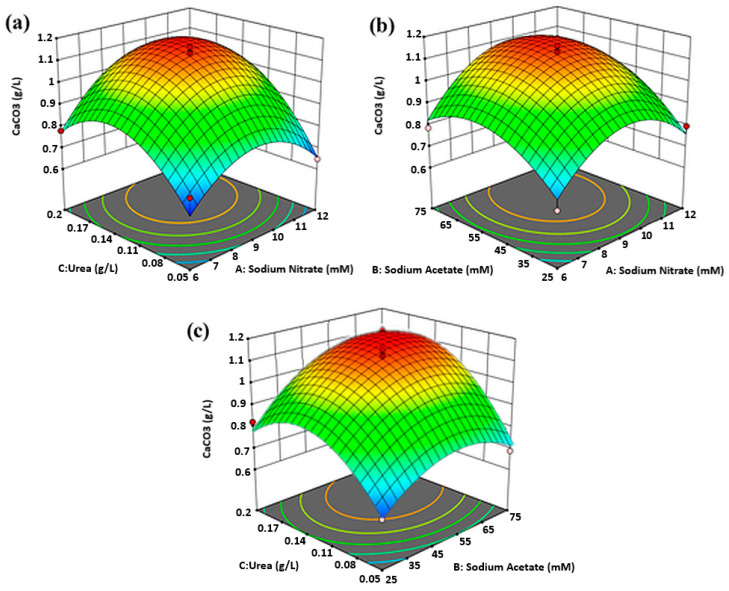
Response surfaces and contour plots showing the mutual effect of (**a**) urea and NaNO_3_, (**b**) sodium acetate and NaNO_3_, and (**c**) urea and sodium acetate on CaCO_3_ precipitates concentration.

Second-order quadratic polynomial equation regressed to the experimental data can be graphically represented by the surface and contour plot as a function of two factors at a time, while keeping the rest constant at their center point. In the present study, the response surface plots of Figure 3a and Figure 4a–c depict a fully developed circular concave surface, thus suggesting the presence of an apparent optimum condition at the peak. In contrast, Figure 3b,c appear to have elliptical contours. The optimum point in these figures might be located at the top-most edge of the incomplete concave plot or perhaps, slightly outside the design boundary. Contour plots are two-dimensional representations of the response surface, which enhance the visual interpretation of the response surface. Response surface showing elliptical contour suggests significant mutual interactions between the independent variables, whereas a circular contour plot implicates negligible interaction between the corresponding variables [21].

According to Figure 3a, the highest biomass density was conceivable when urea was linearly increased to concentration in between 0.125 g/L to 0.155 g/L, with NaNO_3_ modulated to 7.5 mM to 9.0 mM, and sodium acetate held at 50 mM. Alternatively, Figure 3b points to maximum biomass by setting up sodium acetate between 65 mM to 75 mM, and NaNO_3_ from 6.8 mM to 8.5 mM, with urea constant at 0.125 g/L. Figure 3c then shows that maximum biomass production was also observed when NaNO_3_ was maintained constant at 9 mM, while the sodium acetate concentration was maintained from 61.0 mM to 68.5 mM and urea concentration from 0.120 g/L to 0.145 g/L.

The mutual effects of NaNO_3_, urea, and sodium acetate on the concentration of CaCO_3_ precipitates are all shown by the well-defined dome-shaped response surface plots in Figure 4a–c. The effects of urea, NaNO_3_ and their reciprocal interactions on CaCO_3_ precipitation at fixed sodium acetate concentration in Figure 4a shows that CaCO_3_ concentration increased in tandem with urea and NaNO_3_ increasing. By keeping the concentration of sodium acetate constant at 50 mM, maximum CaCO_3_ could be obtained at urea concentration ranging from 0.130 g/L to 0.170 g/L, and NaNO_3_ from 8.8 mM to 10.5 mM. CaCO_3_ precipitation considerably impeded when the concentration of both factors exceeded the stated range. Otherwise, Figure 4b describes the increased in CaCO_3_ concentration when the concentrations of NaNO_3_ and sodium acetate in the BG-11 medium were added at range around 8.5 mM to 10.35 mM and 52 mM to 67 mM, respectively, while maintaining urea concentration at 0.125 g/L. From Figure 4c, augmentation of CaCO_3_ concentration was the result of adding the concentration of urea and sodium acetate between 0.135–0.177 g/L and 54–69 mM, respectively, when NaNO_3_ was maintained at the center point.

### 2.5. Experimental Validation of the Proposed Model 

The optimal values of the three factors were computed by simultaneously solving Equation (1) and (2) through Design Expert. The optimal levels were as follows: 8.74 mM of NaNO_3_, 61.40 mM of sodium acetate, and 0.143 g/L of urea. The theoretical responses for biomass density and CaCO_3_ precipitates concentration as predicted under the abovementioned conditions were 1.535 g/L and 1.150 g/L, respectively, with a desirability value of 0.979. Table 7 shows the statistical analysis of the results obtained from the validation run applied to two independent replicates (*N* = 2). The observed responses for biomass and CaCO_3_ precipitation were 1.517 g/L and 1.143 g/L. Experimental values were well within the 95% confidence interval of the models’ prediction. The proximity of the observed and predicted responses indicates that the regression models were sufficiently accurate and reliable for predicting the biomass production and CaCO_3_ precipitation of *C*. *vulgaris*.

An average biomass density gained at 1.517 g/L points to the capability of this locally isolated *C*. *vulgaris* to undergo trophic switching by assimilating organic carbon source in the form of sodium acetate. At 12 h:12 h light and dark photoperiod, the experiment would closely resemble the natural solar exposure of many photoautotrophs, especially those dwell in open pond types of cultivation. Thus, by comparison, the effect of mixotrophic treatment on the propagation of microalgal biomass would be more pronounced since most obligate photoautotrophs would register an average biomass density in the range of less than 1.00 g/L [9]. The same illumination cycle was also considered to be optimum for the growth of MICP microalgae based on the calcification experiment of Gloeocapsa sp. [6].

The response surface method (RSM) optimization concerning CaCO_3_ precipitation by MICP microalgae was also attempted for other Chlorella species. A more recent study by Irfan et al. [22] took an approach of valorizing cement kiln dust (CKD), a waste by-product of limestone calcination to produce cement, as a substrate for biomineralization by *Chlorella kessleri* using Bold’s Basal Medium (BBM). The authors had identified three factors, namely temperature, pH and experiment time as vital factors influencing the CaCO_3_ calcification process. Their RSM optimization exercise produced a ceiling of 25.18 g of CaCO_3_ by standardizing 50 g waste CKD mixed to 130 mL culture medium. The reported figure was very impressive, considering that very high concentration of calcium ions were initially present in the substrate, which can be anywhere between 30–50% of the total CKD content. The outcome was possibly complemented by a more refined experimental setup since their work had employed continuous aeration of compressed air: CO_2_ mixture at fixed 96% to 4% ratio and a dedicated Grolux illumination system specifically for promoting plant growth. Though the exact calcium content in CKD was not clearly stated, the authors claimed that a maximum of 96% calcium was eventually extracted from CKD to precipitate CaCO_3_. By determining the calcium content initially found in 12 mM CaCl_2_.2H_2_O feedstock (0.481 g/L) and 1.143 g/L CaCO_3_ obtained from the final validation run (0.458 g/L), we can establish that the conversion or biomineralization efficiency of *C. vulgaris* in this study stands at 95.2%, which is very much comparable to the biomineralization output induced by *C. kessleri.*

It was also worth noting that the authors’ best CaCO_3_ yield was achieved at nine days, which was at the onset of stationary phase as similar to *C. vulgaris* used in this study. At various alkaline pH conditions favoring calcite deposition by *C. vulgaris*, Ramanan et al. [5] also observed that the microalga cultivation usually exhibited the highest values of maximum specific growth rate (µ*_max_*) on Day 9–10 (0.06 to 0.08 g/L/day) but µ_max_ then gradually reduced to its lowest on Day 14. Since the performance of CaCO_3_ precipitation was observed to increase concomitantly with the cell growth, the duration of *C. vulgaris* cultivation time was decidedly halted on Day 14 for this study.

From Table 7, the precision of the experimental results was satisfactory with values of the standard deviations for both biomass density, and CaCO_3_ precipitates were within 5% and 6% of the measured averages, respectively. However, as we are dealing with the uncertainties of microbiological system, the breadth of error margin can widely differ from one study to the next. In the cultivation experiments involving five species of Chlorella (*C. vulgaris, C. minutissima, C. pyrenoidosa, Chlorella sp. 1*, and *Chlorella sp. 2*) grown in various nutrient medium (i.e., BG-11, BBM, Fog’s medium, and M_4_N medium) for the assessment of biomass evolution and lipid production potential, the standard deviations in Sharma et al. [23] results ranged from as small as 1.2% up to 4.3%. As for data describing the calcium carbonate precipitation by Chlorella, sometimes it may also be expressed as water hardness (calcium ions) removal from the liquid samples. As per recent findings by Wang et al. [14], the recorded standard deviation of calcium carbonate precipitation, in terms of calcium ions removal by *C. vulgaris*, ranged between 1.64% and 12.53%.

### 2.6. Scanning Electron Microscopy (SEM) and X-Ray Diffraction (XRD) Analyses 

**Figure 5:**
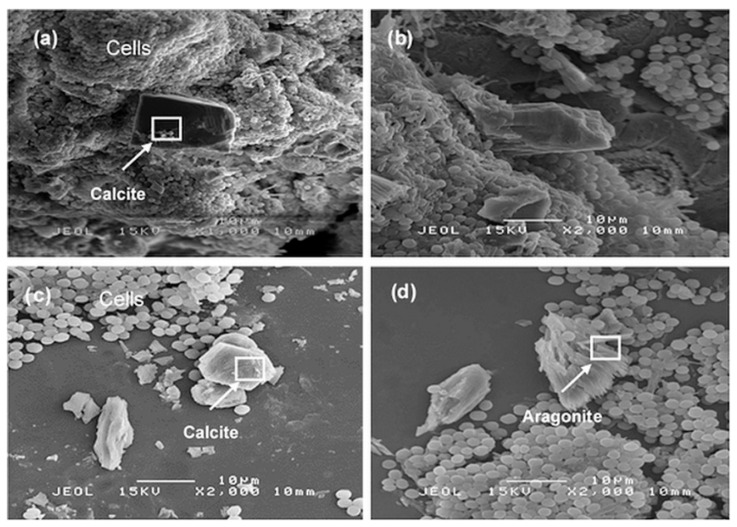
SEM images of CaCO_3_ crystals formed by *C. vulgaris* (**a**) rhombohedric calcite crystal protruding from masses of cells at 1000× magnification, (**b**) side view of crystal protrusion for masses of cells at 2000× magnification, (**c**) free-floating calcite crystals at 2000× magnification, and (**d**) needle-like crystals possibly aragonite at 2000× magnification.

The CaCO_3_ precipitates were viewed under SEM to determine the morphological characteristics of the crystals. SEM magnifications of the CaCO_3_ crystals collected from *C*. *vulgaris* are shown in Figure 5. There is a clear appearance of mineral protrusion emerging from the masses of spherical *C. vulgaris* cells in Figure 5a,b, an association that strongly suggests the cells serving as nucleation sites for CaCO_3_ crystals formed during the MICP process. Figure 5c,d reveal several of the free-floating fragmented crystals. All in all, the majority of CaCO_3_ minerals in SEM images distinctly embody a profile that appears layered and rhombohedric in shape, matching the rhombohedral habit of calcite polymorph [24]. Nonetheless, upon closer inspection of Figure 5d, there also appears to be several needle-like crystals seemingly clumped together, a possible indication to aragonite precipitation as well and potentially being the precursor to subsequent calcite formation.

**Figure 6:**
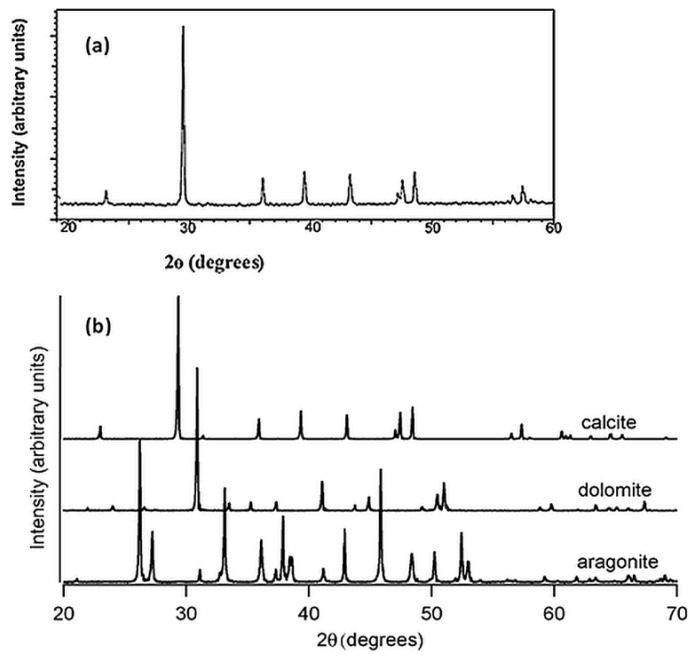
XRD diffractograms of (**a**) CaCO_3_ crystals from this study, (**b**) representative XRD of spar calcite, geogenic dolomite, and spar aragonite.

The crystallographic properties of CaCO_3_ precipitates were then further authenticated by powder X-ray diffraction (XRD) analysis. Figure 6a shows the peak patterns of the crystalline structure of CaCO_3_ minerals obtained from biomineralization activity of *C. vulgaris*. Initially, the finding was compared against spar calcite, geogenic dolomite, and spar aragonite reference diffractograms in Figure 6b. Alignment to the reference peaks shows that phase compositions in Figure 6a displays the highest peak at 29.5°, which conforms to the characteristic XRD peak of spar calcite. Further confirmation was achieved through comparing the tabulated spectra from this study with powder diffraction file (PDF) of related mineral. The same XRD results characteristically produced the strongest detected (*hkl*) peaks at 2*θ* values of 23.1, 29.5, 36.0, 39.5, 43.2, 47.6, 48.6, corresponding to the Miller indices of (012), (104), (110), (113), (202), (018), (116), (122), (214), (300), which are the pure crystallographic planes of calcite, respectively (PDF# 05-586).

It seems that the crystalline properties and morphology of CaCO_3_ are very much influenced by the method of synthesis. *C. vulgaris*, like many photosynthetic microorganisms, follows the “biologically induced-mineralization” (BIM) mechanism whereby CaCO_3_ crystals are precipitated indirectly from the interactions between the metabolic by-products that were secreted out of cells and the inorganic compounds present in the immediate microenvironment. As such, it is more of a diffusion phenomenon that follows calcium supersaturation in which *C. vulgaris* cells have limited control over composition, localization, and nucleation of CaCO_3_ crystals. BIM generated CaCO_3_ precipitates are effectively varied in the size of particulates, crystallinity, and morphology [8]. On the other hand, calcite is widely considered the dominant polymorph in the biomineralization mediated by microalgae. Precipitation of CaCO_3_ minerals started with the formation of unstable amorphous phase around the cells’ locality, which is then transformed to metastable phase aragonite or a lesser degree, vaterite. Further increased in the pH during photosynthesis and inorganic carbon fixation, deemed most crucial factor for the formation of the polymorph in a constant-composition environment, will favor the final crystal transformation to the more thermodynamically stable phase calcite [25].

Other metal ions present in the medium ingredients may also modify the precipitation of different polymorph. It was reported that the presence of Zn^2+^ ions would slightly lower the microenvironment pH surrounding the cells, a condition preferring the building of aragonite [26]. Both Ca^2+^, as well as Zn^2+^ cations, may be adsorbed onto the negatively charged cell walls. The microalgal nucleation sites which adsorbed Zn^2+^ enable local enrichment of this ion, whereby their interaction will stabilize the metastable aragonite modification. Zn^2+^ that strongly adsorbed on aragonite crystals inhibits further transformation to calcite. Mg^2+^ also demonstrated similar inhibiting effect through adsorption onto the specific growth sites on the crystal surface or by increasing the crystal solubility through incorporation into the calcite lattice [27]. In a comparison study between abiotic and biotic system containing *C. vulgaris* cells, aragonite was mostly produced in an abiotic solution containing Mg^2+^ ions of up to 5.0 mM. On the contrary, calcite was preferentially produced in the biotic system under the influence of both photoautotrophic and heterotrophic *C. vulgaris* cultures. The profound affinity of adsorption of exopolysaccharide (EPS) released by *C. vulgaris* on the aragonite surface over calcite (possibly due to the presence of carboxylic compounds in the EPS) was attributed to the stronger inhibition of aragonite polymorph formation despite the presence of Mg^2+^ ions in the medium [13].

## 3. Materials and Methods

### 3.1. Microalgae Strain and Inoculum Preparation 

*Chlorella vulgaris* used throughout this study was originally isolated off the waters of Pulau Sayak Island, Malaysia. Following strain identification, stock culture is maintained by the International Institute of Aquaculture & Aquatic Science, Universiti Putra Malaysia (I-AQUAS UPM), Malaysia. *C. vulgaris* from slant agar were transferred to Blue-Green (BG) 11 aquaculture medium [28] and incubated at 25 °C in an orbital shaker (Ecotron, INFORS-HT, Switzerland) at 110 rpm for 14 days to obtain a cell concentration of approximately 3.3 × 10^7^ cells/mL. The composition of BG-11 in g/L consisted of the following: 1.5 g NaNO_3_, 0.04 g K_2_HPO_4_, 0.075 g MgSO_4_.7H_2_O, 0.036 g CaCl_2_.2H_2_O, 0.006 g citric acid, 0.006 g ferric ammonium citrate, 0.001 g EDTA (disodium salt), 0.02 g Na2CO_3_, and 1 mL of trace metal mix A5 (g/L) (2.86 g H_3_BO_3_, 1.81 g MnCl_2_.4H_2_O, 0.222 g ZnSO_4_.7H_2_O, 0.39 g Na_2_MoO_4_.2H_2_O, 0.079 g CuSO_4_.5H_2_O, 0.049 g Co(NO_3_)_2_.6H_2_O). Illumination was provided through manual installation of T5 fluorescent tubes (OSRAM, Germany) inside the shaker unit, illuminating the cultures at about 2280 ± 250 lux or an equivalent photosynthetic photon flux (PPF) of 30 ± 3.3 μmol/m^2^⋅s for a constant diurnal cycle of 12 h (light): 12 h (dark). The culture was used as standard inoculums for all cultivations in shake flasks.

### 3.2. Selection of Factors in Medium Formulation

Eight factors (NaNO_3_, K_2_HPO_4_, MgSO_4_.7H_2_O, ZnSO_4_.7H_2_O, sodium acetate, urea, temperature, and salinity) were screened for their significances towards growth and MICP-producing capabilities of *C. vulgaris* cultured in BG-11 medium. NaNO_3_, K_2_HPO_4_, MgSO_4_.7H_2_O and ZnSO_4_.7H_2_O are components initially present in BG-11. These existing nitrogen and major ionic macronutrients were chosen for evaluation as to whether their increment or reduction would have a significant effect on the two responses. Their levels were determined based on *Chlorella* cultivation data found in the literature. Sodium acetate served as a carbon source while urea stood for nitrogen source as well as feed material for MICP via urea hydrolysis. The range of urea tolerable by *C*. *vulgaris* strain was previously determined through one-factor-at-a-time (OFAT) experiments [29]. Temperature was the sole exogenous factor chosen. Since *C. vulgaris* is also halotolerant with recognizable desalination ability [30], the impact of salinity was included by adding sodium chloride to mimic the conditions from freshwater to the marine environment. 

In this study, 5 mM of NaHCO_3_ was used as substitute DIC for Na_2_CO_3_ since it is more soluble in water. The supply of Ca^2+^ ions was provided by 12 mM of CaCl_2_.2H_2_O, respectively. Stock solutions of the selected chemicals were added separately and aseptically to 250 mL Erlenmeyer flask and topped up with 85 mL BG-11 medium devoid of chemicals chosen as experimental variables. All mixotrophic cultivations were standardized to 100 mL by adding 15 mL of inoculum (15% *v*/*v*). Using a pH meter (Eutech Instrument, Vernon Hills, IL USA), the pH of the mixture was adjusted to ≈ 7.5 by the addition of either 0.1N HCl or NaOH. The medium was sterilized by autoclaving at 121 °C for 15 min. The cultures were incubated in an orbital incubator shaker agitated at 110 rpm for 14 days. All cultivations were carried out in duplicates.

### 3.3. Biomass Density

The microalgal biomass, expressed as dry cell weight per culture volume (g/L), was determined gravimetrically using the filtration and oven-drying method. After 14 days, 10 mL of cell culture was vacuum filtered through a pre-weighed dried glass-fiber filter (Sartorius, Germany). 10 mL of 50 mM NaHCO_3_ and distilled water were run through the filter twice to wash the cells. Separately, 10 mL of 1N HCl was added to dissolve the CaCO_3_ precipitates lodged to the cell suspension [31]. The filtered cells and filter paper were dried in an oven at 70 °C for 48 h until a constant weight was obtained.

### 3.4. Quantification of CaCO_3_ Precipitation

During the filtration process, the filtrate containing dissolved CaCO_3_ was collected after 10 mL of 1N HCl was run through the filter [31]. The hydrochloric acid reacted with CaCO_3_ to form the water-soluble CaCl_2_, which has a 1:1 molar ratio to CaCO_3_ in terms of Ca^+^ ion concentration. The concentration of Ca^+^ ion obtained was measured by using ethylenediaminetetraacetic acid (EDTA) titration following the APHA standard method [32]. The molarity of Ca^2+^ that was present in the filtrate was calculated, as shown in Equation (3). Molarity of Ca^2+^ was expressed as CaCO_3_ equivalent. Conversion to CaCO_3_ concentration (in g/L) was done by multiplying Ca^2+^ molarity with CaCO_3_ molecular weight (100.0869 g/mol):

(3) $$Ca^{2+}(M)=\frac{\mathrm{molarity}\;\mathrm{of}\;\mathrm{EDTA}\;\times \;\mathrm{titre}\;\mathrm{volume}\;(\mathrm{mL})}{\mathrm{filtrate}\;\mathrm{volume}\;(\mathrm{mL})}$$

### 3.5. Scanning Electron Microscopy 

Before SEM examination, the microalgal samples were centrifuged at 5000 rpm (equivalent to relative centrifugal force of 1845× *g*) for 5 min (Microfuge 16, Beckman Coulter, Brea; CA, USA). The supernatant was decanted, and the pellet was fixed with 2.5% (*v*/*v*) glutaraldehyde for 4 to 6 h at 4 °C. The samples were washed three times, with 0.2% (*v*/*v*) sodium cacodylate buffer for 10 min each. The samples were then post-fixed with 1% (*v*/*v*) osmium tetroxide for 2 h at 4 °C and washed three times with sodium cacodylate buffer for 10 min each. After post-fixation, the samples were dehydrated gradually using a series of increasing acetone concentrations: 35%, 50%, 75%, 95% once for 10 min each, and 100% three times for 15 min each. After the drying process, the samples were mounted on aluminum stubs and coated with gold using a sputter coater. The samples were then observed using SEM (Model JEOL JSM-6400, Tokyo, Japan) with an accelerating voltage of 15.0  kV.

### 3.6. X-ray Diffraction Analysis

The crystalline phase of the CaCO_3_ produced after 14 days was analyzed by powder X-ray diffraction (XRD). The XRD measurements were conducted on a Shimadzu XRD-6000 Diffractometer equipped with a Cu-tube (30 kV, 30 mA). The primary-beam was employed in a θ range of 2° to 60° at 2°/min.

### 3.7. Medium Formulation Through Statistical Design and Modeling

The eight factors considered were screened at high (−1) and low (+1) preset, as shown in Table 8. Design Expert Version 11.0.5 (Stat-Ease Inc., Minneapolis, MN, USA) was used to design the experimental matrices of both Plackett–Burman Design (PBD) and Box–Behnken Design (BBD), performed ANOVA analysis on the dataset, derivation of polynomial model equations, generation of response surface plots and lastly, prediction of the optimum levels of factors. In PBD, the significant factors identified from the half-normal plot analysis were fitted to the first-order model, as shown in Equation (4):

(4) $$Y=B_{0}+\overset{n}{\underset{i=1}{\sum }}B_{i}X_{i}$$

where Y is the predicted response, $B_{0}$ the model constant, $X_{i}$ the independent variable, $B_{i}$ the linear coefficient of the variable. Factors deemed significant were then chosen for response surface optimization by BBD method. The total number of experimental runs (*N*) designed by BBD was based upon the relationship of:

*N* = 2*k*^2^ − 2*k* + C_p_ (5)

where *k* is the factorial number, and C_p_ is the replications at center points [33]. The outcomes of the experiments were regressed to the second-order polynomial function to correlate the relationship existed between the response and the independent variables, as shown in Equation (6):

(6) $$Y=B_{0}+\overset{n}{\underset{i=1}{\sum }}B_{i}X_{i}+\overset{n}{\underset{i<j}{\sum }}B_{ij}X_{i}X_{j}+\overset{n}{\underset{j=1}{\sum }}B_{jj}X_{j}{}^{2}$$

where *Y* is the predicted response; $B_{0}$ is the estimated regression coefficient of the fitted response; $X_{i}$ represents the non-coded independent variables. The regression coefficient for the linear term is represented by $B_{i}$ while $B_{ij}$ the second-order interaction, and $B_{jj}$ the quadratic coefficient.

### 3.8. Model Validation Runs

Experimental runs were carried out in duplicates to confirm the validity of the predicted model. *C. vulgaris* was cultivated in BG-11 medium under the same cultivation conditions as described previously but with the concentrations of significant factors from BBD following the predicted composition having the highest desirability from Design Expert simulation. The cultures were also cultivated for 14 days. The average responses of the biomass and CaCO_3_ concentration would be compared with those predicted by the regression models.

## 4. Conclusions

Plackett–Burman Design was employed in this study to screen the eight factors presumed to contribute significant effect towards biomass density and CaCO_3_ precipitation of microalga *C. vulgaris*. It was found that the two responses were highly dependent on NaNO_3_, urea, and sodium acetate. Fine-tuning the formulation of the three components then made use of the RSM variation of Box–Behnken Design. The maximum biomass density of 1.517 g/L and CaCO_3_ mineral concentration of 1.143 g/L were observed in BG-11 medium formulated with 8.74 mM of NaNO_3_, 61.40 mM of sodium acetate, and 0.143 g/L of urea, respectively, conforming to the RSM prediction at 95% confidence level. SEM analysis and elucidation by XRD indicated that calcite was the dominant CaCO_3_ polymorph formed during the MICP process by *C. vulgaris* cells.

## Figures and Tables

**Table 1:** Plackett–Burman Design of eight factors in actual levels with predicted and observed values of *C. vulgaris* biomass density and CaCO_3_ precipitates.

No.	Factors								Biomass (g/L)		CaCO_3_ (g/L)	
	*A*	*B*	*C*	*D*	*E*	*F*	*G*	*H*	Predicted	Observed	Predicted	Observed
1	22.00	0.100	0.020	0.1	0.07	7.50	30	0.05	0.87	0.85	0.361	0.389
2	22.00	0.100	0.00077	0.1	0.07	50.00	24	30.00	1.00	1.00	0.694	0.655
3	2.50	0.100	0.020	0.1	0.33	50.00	30	0.05	1.25	1.20	0.652	0.624
4	22.00	0.010	0.00077	0.1	0.33	7.50	30	30.00	0.60	0.50	0.206	0.190
5	22.00	0.010	0.020	1.5	0.07	50.00	30	30.00	0.85	0.90	0.561	0.588
6	12.25	0.055	0.01038	0.8	0.20	28.75	27	15.03	1.05	1.10	0.583	0.598
7	2.50	0.010	0.020	0.1	0.33	50.00	24	30.00	0.98	1.00	0.625	0.654
8	22.00	0.100	0.00077	1.5	0.33	50.00	24	0.05	1.12	1.20	0.756	0.795
9	2.50	0.100	0.00077	1.5	0.33	7.50	31	30.00	1.25	1.30	0.703	0.720
10	2.50	0.100	0.020	1.5	0.07	7.50	24	30.00	1.25	1.20	0.830	0.813
11	2.50	0.010	0.00077	1.5	0.07	50.00	30	0.05	1.10	1.00	0.717	0.690
12	22.00	0.010	0.020	1.5	0.33	7.50	24	0.05	0.72	0.70	0.396	0.356
13	2.50	0.010	0.00077	0.1	0.07	7.50	24	0.05	0.85	1.00	0.490	0.517
14	12.25	0.055	0.01038	0.8	0.20	28.75	27	15.03	1.05	1.00	0.583	0.568

*A* = NaNO_3_ (mM), *B* = urea (g/L), *C* = ZnSO_4_.7H_2_O (mM), *D* = MgSO_4_.7H_2_O (mM), *E* = K_2_HPO_4_ (mM), *F* = sodium acetate (mM), *G* = temperature (°C), *H* = salinity (ppt).

**Table 2:** ANOVA of the reduced model from Plackett–Burman design (PBD) screening for *C. vulgaris* biomass density.

Source	Sum of Squares	df	Mean Square	F-Value	*p*-Value
Model	0.5208	4	0.1302	16.08	0.0007
*A-Sodium nitrate*	0.2002	1	0.2002	24.72	0.0011
*B-Urea*	0.2269	1	0.2269	28.01	0.0007
*D-Magnesium sulfate*	0.0469	1	0.0469	5.79	0.0428
*F-Sodium acetate*	0.0469	1	0.0469	5.79	0.0428
*Curvature*	0.0067	1	0.0067	0.8268	0.3898
Residual	0.0648	8	0.0081		
Lack of Fit	0.0598	7	0.0085	1.71	0.5308
Pure Error	0.0050	1	0.0050		
Corrected Total	0.5923	13			

Model summary: *R*^2^ = 0.8894; Coefficient of Variation (C.V.) = 9.03%; Adjusted-*R*^2^ (Adj-*R*^2^) = 0.8340; Predicted-*R*^2^ (*Pred*-R^2^) = 0.6658; df, degrees of freedom.

**Table 3:** ANOVA of the reduced model from PBD screening for CaCO_3_ precipitates concentration.

Source	Sum of Squares	df	Mean Square	F-Value	*p*-Value
Model	0.36	5	0.073	19.82	0.0005
A-Sodium nitrate	0.091	1	0.091	24.77	0.0016
B-Urea	0.084	1	0.084	22.82	0.0020
D-Magnesium sulfate	0.073	1	0.073	19.88	0.0029
F-Sodium acetate	0.087	1	0.087	23.72	0.0018
G-Temperature	0.029	1	0.029	7.91	0.0260
Curvature	5.131 × 10^−8^	1	5.131 × 10^−8^	1.401 × 10^−5^	0.9971
Residual	0.026	7	3.661 × 10^−3^		
Lack of Fit	0.025	6	4.198 × 10^−3^	9.57	0.2425
Pure Error	4.387 × 10^−4^	1	4.387 × 10^−4^		
Corrected Total	0.39	13			

Model summary: *R*^2^ = 0.9340; C.V. = 9.72%; Adj-*R*^2^ = 0.8928; Pred-*R*^2^ = 0.7512.

**Table 4:** Three-factor Box–Behnken Design (BBD) with factorial levels shown in actual values as well as predicted and observed biomass density and CaCO_3_ precipitates concentration.

Run Order	NaNO_3_ (mM)	Sodium Acetate (mM)	Urea (g/L)	Biomass Density (g/L)		CaCO_3_ Precipitates (g/L)	
				Predicted	Observed	Predicted	Observed
1	9	50	0.125	1.49	1.50	1.121	1.162
2	9	25	0.200	1.15	1.10	0.784	0.824
3	12	50	0.050	0.91	0.85	0.654	0.648
4	12	25	0.125	1.01	1.10	0.759	0.796
5	12	50	0.200	0.94	0.90	0.988	0.910
6	12	75	0.125	1.04	1.05	0.997	1.043
7	9	75	0.200	1.26	1.30	1.072	1.103
8	9	75	0.050	1.30	1.35	0.728	0.688
9	6	50	0.050	1.06	1.10	0.634	0.712
10	9	50	0.125	1.49	1.55	1.121	1.135
11	6	50	0.200	1.29	1.35	0.775	0.781
12	9	50	0.125	1.49	1.50	1.121	1.133
13	9	25	0.050	0.84	0.80	0.653	0.622
14	6	75	0.125	1.54	1.45	0.824	0.787
15	9	50	0.125	1.49	1.43	1.121	1.117
16	6	25	0.125	1.01	1.00	0.699	0.652
17	9	50	0.125	1.49	1.45	1.121	1.056

**Table 5:** ANOVA for the quadratic model of dry cell weight of *C. vulgaris*.

Source	Sum of Squares	df	Mean Square	F-Value	*p*-Value
Model	0.9447	9	0.1050	16.28	0.0007
A-Sodium nitrate	0.1250	1	0.1250	19.39	0.0031
B-Sodium acetate	0.1653	1	0.1653	25.64	0.0015
C-Urea	0.0378	1	0.0378	5.87	0.0460
AB	0.0625	1	0.0625	9.70	0.0170
AC	0.0100	1	0.0100	1.55	0.2530
BC	0.0306	1	0.0306	4.75	0.0657
A^2^	0.1879	1	0.1879	29.15	0.0010
B^2^	0.0645	1	0.0645	10.00	0.0159
C^2^	0.2108	1	0.2108	32.70	0.0007
Residual	0.0451	7	0.0064		
Lack of Fit	0.0356	3	0.0119	5.00	0.0770
Pure Error	0.0095	4	0.0024		
Corrected total	0.9899	16			

Model summary: *R*^2^ = 0.9544; C.V. = 6.57%; Adj-*R*^2^ = 0.8958; Pred-*R*^2^ = 0.4092; Adequate Precision = 11.4689; df, degrees of freedom.

**Table 6:** ANOVA for the quadratic model of CaCO_3_ concentration as precipitated by *C. vulgaris*.

Source	Sum of Squares	df	Mean Square	F-Value	*p*-Value
Model	0.6057	169	0.0673	15.35	0.0008
A-Sodium nitrate	0.0270	1	0.0270	6.17	0.0420
B-Sodium acetate	0.0661	1	0.0661	15.07	0.0060
C-Urea	0.1123	1	0.1123	25.63	0.0015
AB	0.0031	1	0.0031	0.7154	0.4256
AC	0.0093	1	0.0093	2.12	0.1883
BC	0.0113	1	0.0113	2.59	0.1517
A^2^	0.1272	1	0.1272	29.01	0.0010
B^2^	0.0682	1	0.0682	15.57	0.0056
C^2^	0.1426	1	0.1426	32.54	0.0007
Residual	0.0307	7	0.0044		
Lack of Fit	0.0244	3	0.0081	5.20	0.0725
Pure Error	0.0063	4	0.0016		
Corrected total	0.6364	16			

Model summary: *R*^2^ = 0.9518; C.V. = 7.42%; Adj-*R*^2^ = 0.8898; Pred-*R*^2^ = 0.3706; Adequate Precision = 9.5752; df, degrees of freedom.

**Table 7:** Statistical analysis of validation experiments.

Response	Predicted (g/L)	Observed (g/L)	Standard Deviation	Standard Error of Prediction	95% Predicted Interval Low	95% Predicted Interval High
Dry cell weight	1.535	1.517	0.080	0.058	1.397	1.672
CaCO_3_ concentration	1.150	1.143	0.066	0.048	1.037	1.264

**Table 8:** Levels of factors used in Plackett–Burman Design.

Factors	Symbol	Actual Levels of Coded Factors		
		−1	0	+1
NaNO_3_ (mM)	A	2.5	12.25	22
Urea (g/L)	B	0.01	0.055	0.1
ZnSO_4_.7H_2_O (mM)	C	0.00077	0.01039	0.02
MgSO_4_.7H_2_O (mM)	D	0.1	0.8	1.5
K_2_HPO_4_ (mM)	E	0.07	0.2	0.33
Sodium acetate (mM)	F	7.5	28.75	50
Temperature (°C)	G	24	27.5	30
Salinity (ppt)	H	0.05	15.05	30
